# Reported Retinal Detachment in Anti-VEGF-Treated Retinopathy of Prematurity: A Systematic Review and Meta-Analysis

**DOI:** 10.3390/jcm15197598

**Published:** 2026-09-30

**Authors:** Guina Liu, Guojiao Song, Siqi Wang, Wei Xu, Hanqing Zhao, Yunhan Tao, Wenbin Wei, Xiaoshuang Jiang, Hongtao Liu

**Affiliations:** 1 Department of Ophthalmology, West China Second University Hospital, Sichuan University, Chengdu 610041, China; liuguina@scu.edu.cn (G.L.); xuwei2025@scu.edu.cn (W.X.); tao_yunhan@scu.edu.cn (Y.T.); 2 Key Laboratory of Birth Defects and Related Diseases of Women and Children ( Sichuan University), Ministry of Education, Chengdu 610041, China; adaliawang@stu.scu.edu.cn; 3 Beijing Tongren Eye Center, Beijing Key Laboratory of Intraocular Tumor Diagnosis and Treatment, Beijing Tongren Hospital, Capital Medical University, Beijing 100730, China; songuojiao@stu.scu.edu.cn (G.S.); zhq1214@126.com (H.Z.); weiwenbintr@163.com (W.W.); 4 Beijing Ophthalmology & Visual Science Key Lab, Beijing Tongren Hospital, Capital Medical University, Beijing 100730, China; 5 Department of Ophthalmology, West China Hospital, Sichuan University, Chengdu 610041, China; 6 Department of Ophthalmology, Xizang Hospital of West China Hospital, Sichuan University, Lhasa 857000, China

**Keywords:** retinal detachment, retinopathy of prematurity, anti-VEGF, risk factor, systematic review, meta-analysis

## Abstract

**Objectives**: To estimate the incidence of reported retinal detachment (RD) after primary anti-vascular endothelial growth factor (anti-VEGF) treatment for retinopathy of prematurity (ROP) and to explore study-level factors associated with RD incidence. **Methods**: PubMed, EMBASE, Cochrane Library, and Web of Science were searched up to 24 May 2026, for English-language studies reporting RD outcomes after anti-VEGF monotherapy for ROP. Pooled incidence was estimated with a random-effects model using Freeman–Tukey double arcsine transformation. Heterogeneity, subgroup differences, publication bias, and leave-one-out sensitivity were assessed. **Results**: Twenty-seven studies (5598 eyes; 135 RD events) were included, comprising twelve case series, thirteen cohort studies, and two randomized controlled trials; thus, the evidence was predominantly retrospective. The pooled RD incidence was 1.4% (95% CI, 0.5–2.7%; *I*^2^ = 80.8%). The estimate remained stable on leave-one-out sensitivity analysis; the exclusion of Tong et al. markedly reduced heterogeneity. Higher RD incidence was observed in Asian studies, single-center studies, and studies with predominantly Zone I ROP or aggressive ROP (AROP). Incidence varied by postmenstrual age at treatment, gestational age, birth weight, and anti-VEGF agent. No significant publication bias was detected. **Conclusions**: RD after primary anti-VEGF therapy for ROP is uncommon but sight-threatening, with higher incidence associated with greater disease severity and postmenstrual age. Prolonged, risk-stratified follow-up is warranted for infants with Zone I or AROP. Standardized RD definitions, subtype classification, and severity-adjusted prospective comparisons are needed.

## 1. Introduction

Retinopathy of prematurity (ROP) is a proliferative retinal vascular disease that remains a significant but potentially preventable cause of childhood visual impairment worldwide, with a disproportionate burden in less-developed countries [1,2,3,4]. Intravitreal anti-vascular endothelial growth factor (anti-VEGF) therapy has become an important treatment option for severe ROP, particularly for Zone I disease, aggressive ROP (AROP), and cases in which laser photocoagulation is difficult or undesirable [5,6,7,8]. However, retinal detachment (RD) after anti-VEGF therapy has been reported in clinical trials and real-world studies [9,10,11], with incidence estimates varying widely from 0% among infants treated before 36 weeks’ postmenstrual age to 3.4% in higher-risk cohorts [11,12,13]. This variability highlights the difficulty in distinguishing treatment-related effects from the risk conferred by baseline disease severity, as anti-VEGF therapy is preferentially used in eyes with the highest intrinsic risk of unfavorable anatomical progression [14,15]. Moreover, although individual studies have described RD after anti-VEGF therapy, the available evidence has not been systematically synthesized. Therefore, the aim of this systematic review and meta-analysis was to estimate the reported incidence of RD among infants with ROP treated primarily with anti-VEGF agents and to explore study-level factors associated with variation in RD incidence.

## 2. Materials and Methods

This systematic review and meta-analysis was designed, conducted and reported in accordance with the preferred reporting items for systematic reviews and meta-analyses (PRISMA) guidelines (Page et al., 2021, pp. 178–189) [16]. The systematic search, assessment of eligibility, evaluation of the quality, extraction of data and analysis of data have been performed based on predesigned protocol. The protocol of this systematic review and meta-analysis has been registered on the PROSPERO website with the ID number CRD42024555731.

### 2.1. Data Source and Selection Process

For this systematic review and meta-analysis, PubMed, EMBASE, Cochrane Library and Web of Science were searched for relevant studies published in English until 24 May 2026. The keywords included ROP, anti-VEGF and RD. Details of the selection process can be found in Supplementary Material S1 and Section SA.

### 2.2. Eligibility Criteria and Study Selection

Retrieved studies were included in this review if they satisfied the following criteria: (i) the study participants were preterm infants diagnosed with ROP but no pre-existing RD before therapy; (ii) anti-VEGF agents were used as the primary treatment; (iii) the outcomes of the injection exactly included RD; (iv) sufficient data on the incidence and details of RD were explicitly documented; (v) no additional treatments other than anti-VEGF therapy were administered at baseline.

The exclusion criteria were as following: (i) the study population did not include patients with ROP, or RD was not reported as an outcome; (ii) RD occurred prior to the initiation of treatment; (iii) anti-VEGF therapy was not used as the primary treatment, or was combined with other interventions (e.g., laser photocoagulation, cryotherapy, or surgery) at baseline; (iv) insufficient data were available to extract the incidence or clinical characteristics of RD; (v) non-original studies, including reviews (systematic reviews and meta-analyses), editorials, letters, conference abstracts, books, case reports, or case series with a small sample size (e.g., *n* < 10); (vi) duplicate publications or studies with overlapping populations, in which case the most comprehensive or most recent study was included; (vii) animal studies, in vitro studies, or bioinformatics analyses; (viii) studies with insufficient follow-up duration to adequately assess the occurrence of RD; and (ix) studies not published in English.

### 2.3. Methods for Data Extraction

The relevant data was extracted by two independent reviewers from all included studies. The following study setting information was collected for each study: study design, instrument, publication year, main location (including country or region), number of clinical centers, and sample size (number of treated eyes and number of eyes developing RD after treatment). Detailed baseline characteristics of the study population were also extracted, including gestational age (GA), birth weight (BW), postmenstrual age (PMA) at the time of the first injection and the specific diagnosis of ROP. Information on anti-VEGF therapy was also recorded, including the type of agents, route of administration, dosage, duration of follow-up and salvage treatment if the first treatment failed. Information on the recurrence of ROP was also extracted when reported. Given the variability in definitions of recurrence across studies, the specific definition of recurrence used in each study was recorded in detail. If relevant data were not directly available, attempts were made to contact the corresponding authors of the included studies for additional information.

### 2.4. Risk of Bias Assessment

The methodological quality of the included studies was assessed using the Joanna Briggs Institute (JBI) Critical Appraisal tools [17]. Specifically, the JBI critical appraisal checklists for case series and for cohort studies were applied according to the study design. For the randomized controlled trial (RCT), the Cochrane Risk of Bias tool version (RoB) was used [18]. Quality assessment and visualization were performed using R software (version 4.4.2; R Foundation for Statistical Computing, Vienna, Austria). For studies assessed using the JBI tools, each item was rated as “yes”, “no” or “unclear”, in accordance with the JBI guidelines. For the RCT, the RoB 1 tool was used to evaluate the risk of bias across the standard domains. Two reviewers independently evaluated each study, and any disagreements were resolved through discussion or consultation with a third reviewer.

### 2.5. Subgroup Analysis

Subgroup analyses were performed by stratifying the included studies based on several characteristics, including (i) geographic location (Europe, Asia, Africa or North America); (ii) study design (single center vs. multiple centers); (iii) mean GA (≤27 vs. >27 w); (iv) mean BW (≤1000 vs. >1000 g); (v) predominant ROP zone (Zone I/AROP vs. Zone II); (vi) PMA at first injection (≤36 vs. >36 w); (vii) anti-VEGF agents (IVA—aflibercept, IVB— bevacizumab, IVC—conbercept, IVR—ranibizumab, as well as unspecified anti-VEGF therapies); and (viii) number of anti-VEGF drugs involved (single drug vs. multiple drugs).

### 2.6. Statistical Analysis

The primary outcome focused on the incidence of RD, which was pooled and presented using forest plots. All statistical analyses were performed using R software (version 4.4.2; R Foundation for Statistical Computing, Vienna, Austria). Given the anticipated clinical and methodological heterogeneity across studies, a random-effects model was applied to estimate the pooled incidence. To stabilize the variance of proportions, the Freeman–Tukey double arcsine transformation (PFT) was applied for the meta-analysis of incidence data. Studies with zero events were included, and appropriate statistical adjustments inherent to the transformation were used to handle zero-event data.

The *I*^2^ statistics and Cochran’s Q test were used to assess the proportion of total variation due to heterogeneity. Heterogeneity among studies was assessed using Cochran’s Q test and quantified by the *I*^2^ statistic. Sensitivity analyses were conducted by sequentially excluding each study (leave-one-out analysis) to evaluate the robustness and stability of the pooled estimates.

## 3. Results

### 3.1. Selection and Data Extraction

**Figure 1:**
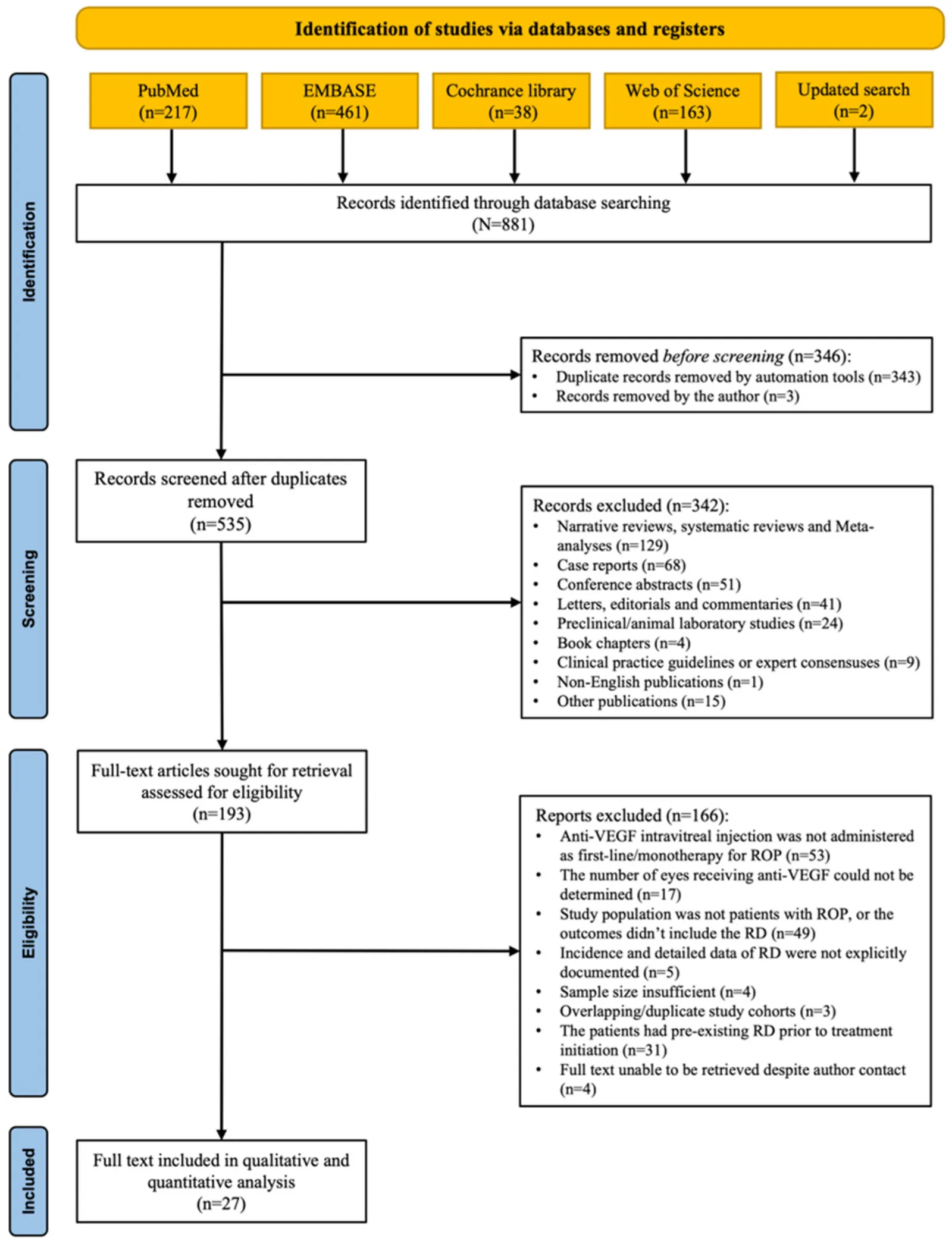
The PRISMA flow diagram detailing the literature search and selection.

A systematic search identified 881 studies based on four databases. After removing 346 duplicates, 535 articles remained for title and abstract screening, of which 342 were excluded for not meeting the inclusion criteria. The remaining 193 articles underwent full-text assessment for eligibility. Next, 166 were excluded for the following reasons: 53 did not use intravitreal anti-VEGF therapy as the primary and sole treatment; 17 lacked clear data on treated eyes; 49 did not include patients with ROP or failed to report RD outcomes; 5 did not provide sufficient data on RD incidence; 4 had insufficient sample sizes; 3 included overlapping cohorts; and 4 were unavailable in full text. Furthermore, 31 studies were excluded for including patients with pre-existing RD before anti-VEGF treatment. Ultimately, 27 studies were included in the qualitative and quantitative meta-analysis (Figure 1), and all of them can be found in Supplementary Material S1.

### 3.2. Characteristics of Included Studies

The key characteristics of included studies are summarized in Table 1. All studies were designed retrospectively and published from 2011 to 2026, with the sample size ranging from 22 patients in the United States to 1224 patients in China. Geographically, the included studies were conducted worldwide, predominantly in Asia (44.5%, *n* = 12), followed by Europe (29.6%, *n* = 8), North America (22.2%, *n* = 6), and Africa (3.7%, *n* = 1). ROP was primarily diagnosed using RetCam (Clarity Medical Systems) or indirect ophthalmoscopy.

Across the included studies, a total of 5598 eyes underwent anti-VEGF therapy. All included ROP patients had a mean GA of 23.2–30.0 weeks and a mean BW of 567.0–1390.5 g. The mean PMA at anti-VEGF treatment ranged from 34.0 to 40.0 weeks, although this information was unavailable in Bazvand, Murakami and Wallace’s study [19]. For subsequent analysis, these cases were stratified according to disease severity based on retinal zone classification. More severe forms, including Zone I ROP and AROP, accounted for 1891 eyes (29.8%). The majority of cases, 3523 eyes (62.9%), were diagnosed as Zone II ROP. A small number of eyes could not be clearly categorized: 32 eyes lacked sufficient information for classification as Zone I ROP and AROP or Zone II ROP. Additionally, 20 eyes were reported as having unclear classification in Xu’s study [20], while Kang’s study reported 12 eyes with Zone III ROP [21]. Another two studies lacked precise zone classification data for 152 eyes [13,22].

To ensure analytical rigor, only eyes with clearly defined diagnostic classifications were extracted and analyzed in subgroup analyses. Details of anti-VEGF therapy are summarized in Supplementary Material S1 Table S2. Among 5598 eyes receiving anti-VEGF therapy, 135 RD events were reported (Supplementary Material S1 Table S3). Details of anti-VEGF therapy are summarized in Supplementary Material S1 Table S2. Four distinct anti-VEGF agents were utilized across the included studies: bevacizumab (3064 eyes, 54.7%), ranibizumab (1941 eyes, 34.6%), conbercept (351 eyes, 6.3%), and aflibercept (242 eyes, 4.4%). The most frequently reported doses were 0.625 mg/0.025 mL for bevacizumab, 0.25 mg/0.025 mL for ranibizumab and conbercept, and 1 mg/0.025 mL for aflibercept. All studies employed intravitreal injection as the route of administration, with the majority using a single-injection regimen. Salvage treatments, including repeat anti-VEGF injections, laser photocoagulation, or vitrectomy, were reported in several studies. The duration of follow-up varied substantially across studies, ranging from 8 weeks to over 2 years.

**Table 1:** Baseline characteristics of 27 included studies.

Study	Study Design	Instruments	Location	Centers	GA (Mean ± SD)	BW (Mean ± SD)	PMA at Therapy (Mean ± SD)	ROP (Zone I) and AROP vs. ROP (Zone II)
Wu, W.C. 2013 [23]	retrospective	RetCam	China	4	26.3 ± 2.6	930.1 ± 359.3	36.6 ± 3.2	17:145
Kang, H.G. 2018 [21]	retrospective	RetCam	Korea	2	27.3 ± 2.5	1049.2 ± 411.1	40.0 ± 2.3	34:107 ^1^
Jang, J.H. 2024 [24]	retrospective	RetCam	Korea	1	26.3 ± 1.8	793.5 ± 195.1	37.7 ± 5.5	17:26
Xu, L.T. 2021 [20]	retrospective	RetCam	USA	1	24.4 ± 0.4	623.1 ± 53.6	35.7 ± 0.7	157:34 ^2^ (other: 20)
Bassiouny, Rm. 2021 [25]	retrospective	RetCam III	Egypt	1	30.0 ± 2.5	1290.0 ± 355.2	38.0 ± 3.1	216:0
Linghu, D. 2022 [26]	retrospective	RetCam	China	1	28.9 ± 2.0	1269.5 ± 371.0	39.0 ± 3.9	262:962
Kabatas, E.U. 2017 [27]	retrospective	IO ^3^	Turkish	1	26.1 ± 2.0	840.7 ± 214.8	34.8 ± 1.8	8:28
Erol, M.K. 2015 [28]	retrospective	IO	Turkish	1	26.7 ± 2.6	895.0 ± 118.2	34.9 ± 1.5	12:24
Ekinci, D.Y. 2021 [29]	retrospective	IO	Turkish	2	28.5 ± 2.7	1167.7 ± 412.8	35.5 ± 2.21	40:34
Bai, Y.C. 2019 [30]	retrospective	IO	China	1	28.5 ± 1.6	1209.6 ± 228.6	34.2 ± 1.9	13:35
Barry, G.P. 2021 [11]	prospective	IO	USA	41	24.6 ± 1.7	657.9 ± 170.8	35.8 ± 2.3	79:85
Ling, K.P. 2020 [31]	retrospective	RetCam III	China	1	26.4 ± 2.2	847.5 ± 234.1	36.0 ± 2.7	32:247
Nicoară, S.D. 2015 [32]	retrospective	RetCam	Romania	1	28.9 ± 1.9	1218.4 ± 326.3	35.11	74:0
Tong, Q.Z. 2018 [33]	retrospective	IO	China	1	30.0 ± 2.0	1340.0 ± 334.0	36.6 ± 2.0	160:0
Çömez, A. 2021 [34]	retrospective	IO	Turkey	2	28.2 ± 2.7	1133.7 ± 356.0	34.0 ± 2.1	112:145
Huang, Q.J. 2017 [35]	retrospective	RetCam	China	1	29.3 ± 2.1	1390.5 ± 412.3	36.0 ± 2.3	150:133
Hwang, C.K. 2015 [36]	retrospective	IO	USA	1	24.2 ± 1.0	668.1 ± 127.3	35.1 ± 2.2	16:6
Jin, E.Z. 2022 [37]	retrospective	RetCam II	China	1	29.49 ± 1.37	1369.0 ± 161.9	38.5 ± 2.7	14:34
Mintz-Hittner, H.A. 2011 [12]	prospective	RetCam	USA	15	24.4 ± 1.2	656.6 ± 129.0	35.2 ± 1.9	66:84
Lundgren, P. 2021 [13]	retrospective	IO	Sweden	7	23.2 ± 0.5	567.0 ± 133.5	34.1 ± 3.8	NA
Bazvand, F. 2021 [19]	retrospective	IO	Iran	1	28.0 ± 2.0	1121.0 ± 312.0	NA	187:678
Hejkal, T.W. 2025 [22]	retrospective	IO	USA	4	25.0 ± 1.3	655.0 ± 176.0	36.0 ± 3.0	NA
Yetik, H. 2015 [38]	retrospective	IO	Turkey	1	28.2 ± 1.9	1005.5 ± 305.9	34.5 ± 1.9	140:98
Akıdan, M. 2026 [10]	retrospective	IO	Turkey	1	27.6 ± 2.2	922.6 ± 199.8	40.0 ± 5.5	45:504
Wallace, D.K. 2018 [39]	RCT	IO	USA	12	24.9	709.0	NA	26:32
Murakami, T. 2021 [40]	retrospective	IO	Japan	1	26.8 ± 2.9	816.0 ± 369.0	NA	4:20
Chen, S.N. 2015 [41]	retrospective	IO	China	2	26.4 ± 0.51	860.3 ± 62.6	36.6 ± 1.0	10:62

^1^ The original research reported that 12 eyes were diagnosed with zone III ROP. ^2^ Twenty eyes were diagnosed with ROP not confined to Zone I or Zone II in the original studies, with no explicit description of the exact subtypes. ^3^ IO: indirect ophthalmoscope. NA: not available.

### 3.3. Results of Quality Assessment

**Figure 2:**
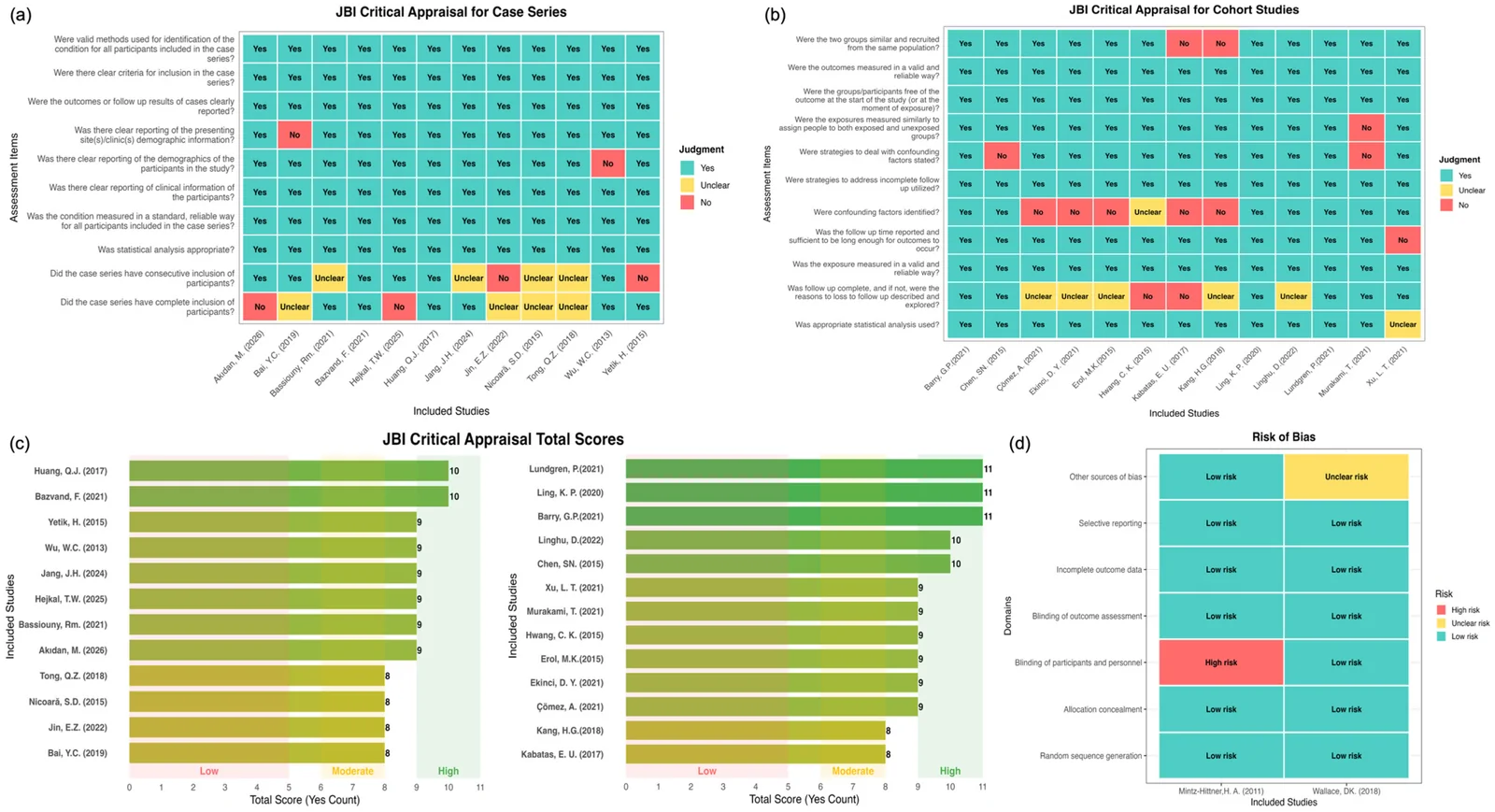
Methodological quality and risk-of-bias assessments of all eligible studies. (**a**) JBI Critical Appraisal Checklist ratings for included case series studies. Each column corresponds to an individual included case series publication. (**b**) JBI Critical Appraisal Checklist ratings for included cohort studies, with identical color grading criteria as panel (**a**). (**c**) Total affirmative “Yes” scores from JBI appraisal for case series (**left**) and cohort studies (**right**). Studies were categorized as low, moderate, or high methodological quality by total Yes counts. (**d**) Risk-of-bias summary plot for the two randomized controlled trials (RCTs) in the dataset across standard Cochrane bias domains.

The methodological quality of the included studies was assessed according to study design, comprising 12 case series, 13 cohort studies, and 2 RCT studies (Figure 2). For case series, most JBI items were fulfilled, particularly in condition identification, outcome measurement, and statistical analysis. However, limitations were noted in demographic reporting, consecutive inclusion, and the completeness of participant recruitment. Cohort studies showed overall moderate to high quality, with similar weaknesses in participant selection and reporting. Overall, cohort studies were predominantly of high quality, while case series were mainly of moderate to high quality. The two included RCTs were judged to have a low risk of bias across most domains. A high risk of bias for the blinding of participants and personnel was identified in one RCT, while the other RCT was rated as unclear for other sources of bias.

### 3.4. The Incidence of RD After Anti-VEGF Therapy

**Figure 3:**
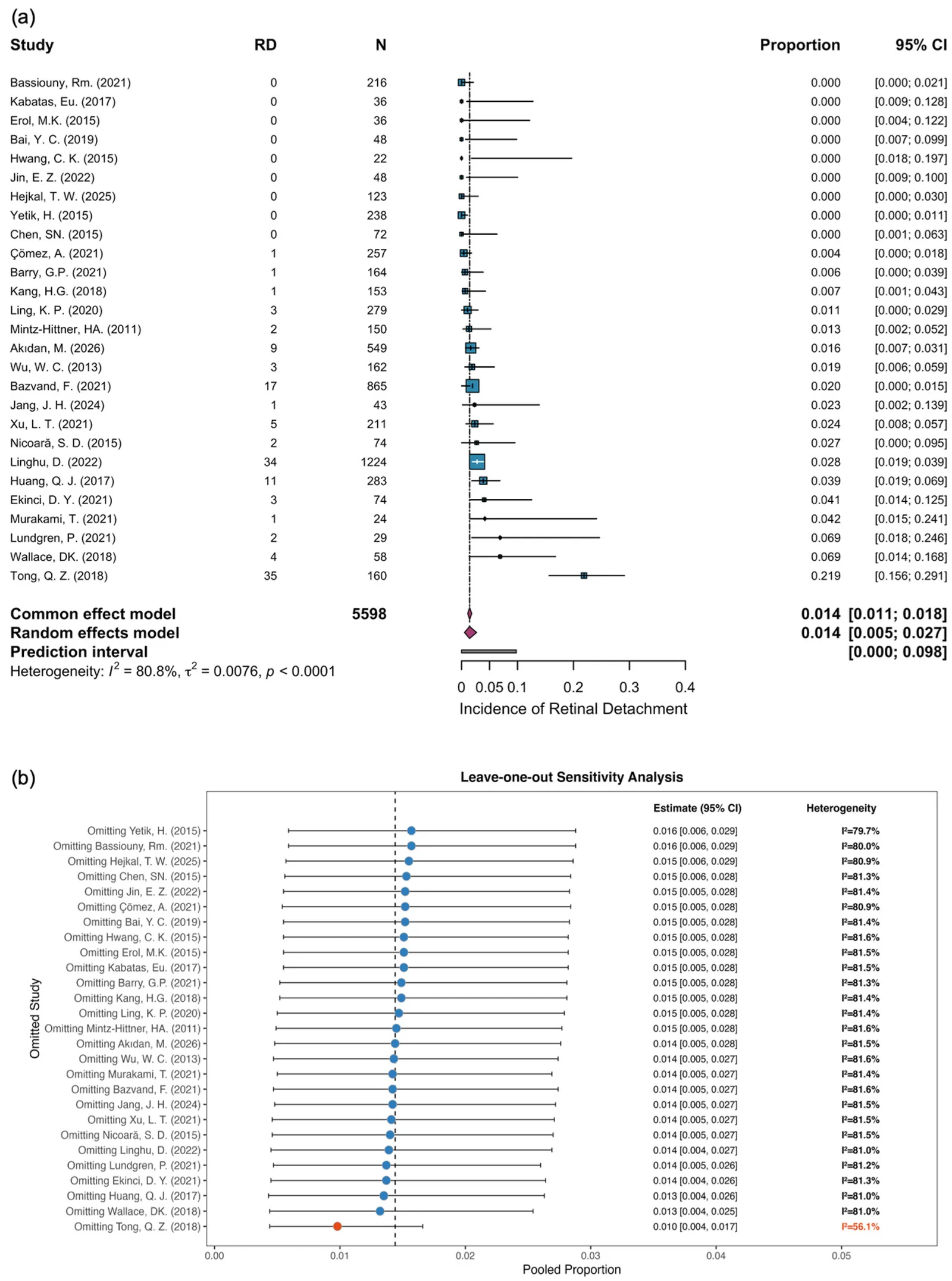
The incidence of retinal detachment (RD) of ROP pre-term infants after anti-VEGF therapy. (**a**) A forest plot of the effect sizes (ES) with 95% confidence intervals in individual studies and overall pooled effect (random effects model); (**b**) the leave-one-out sensitivity analysis. The blue circle and horizontal lines represent the pooled incidence and corresponding 95% CI after omitting each individual study. The red circle and text show the pooled proportion and heterogeneity upon excluding Tong QZ et al. (2018) [33], whose removal substantially changed the pooled estimate and heterogeneity.

Substantial heterogeneity (*I*^2^ = 80.8%, *p* < 0.0001) was detected among the included studies in Figure 3. Thus, a random-effects model was applied for meta-synthesis, yielding a pooled incidence of reported RD of 1.4% (95% CI, 0.5–2.7%) among preterm infants with ROP receiving anti-VEGF injection. Leave-one-out sensitivity analysis demonstrated that the overall pooled estimate remained robust, with recalculated proportions ranging from 0.010 to 0.016. Notably, the exclusion of the study by Tong et al. [33] resulted in a marked reduction in heterogeneity (*I*^2^ = 56.1%), indicating that this study was a major source of heterogeneity. However, the synthesized pooled estimate remained largely unchanged (1.0%, 95% CI 0.4–1.7%, Supplementary Material S1), confirming the robustness of the pooled incidence of reported RD.

### 3.5. Results of Subgroup Analysis

The distribution of studies according to the method of subgroup analysis was as follows: 14 studies had a mean GA ≤ 27 weeks, 15 had a mean BW ≤ 1000 g, 15 had a mean PMA ≤ 36 weeks. Additionally, 18 studies were single-center, and 17 had a predominance of Zone II cases (Table 2).

**Figure 4:**
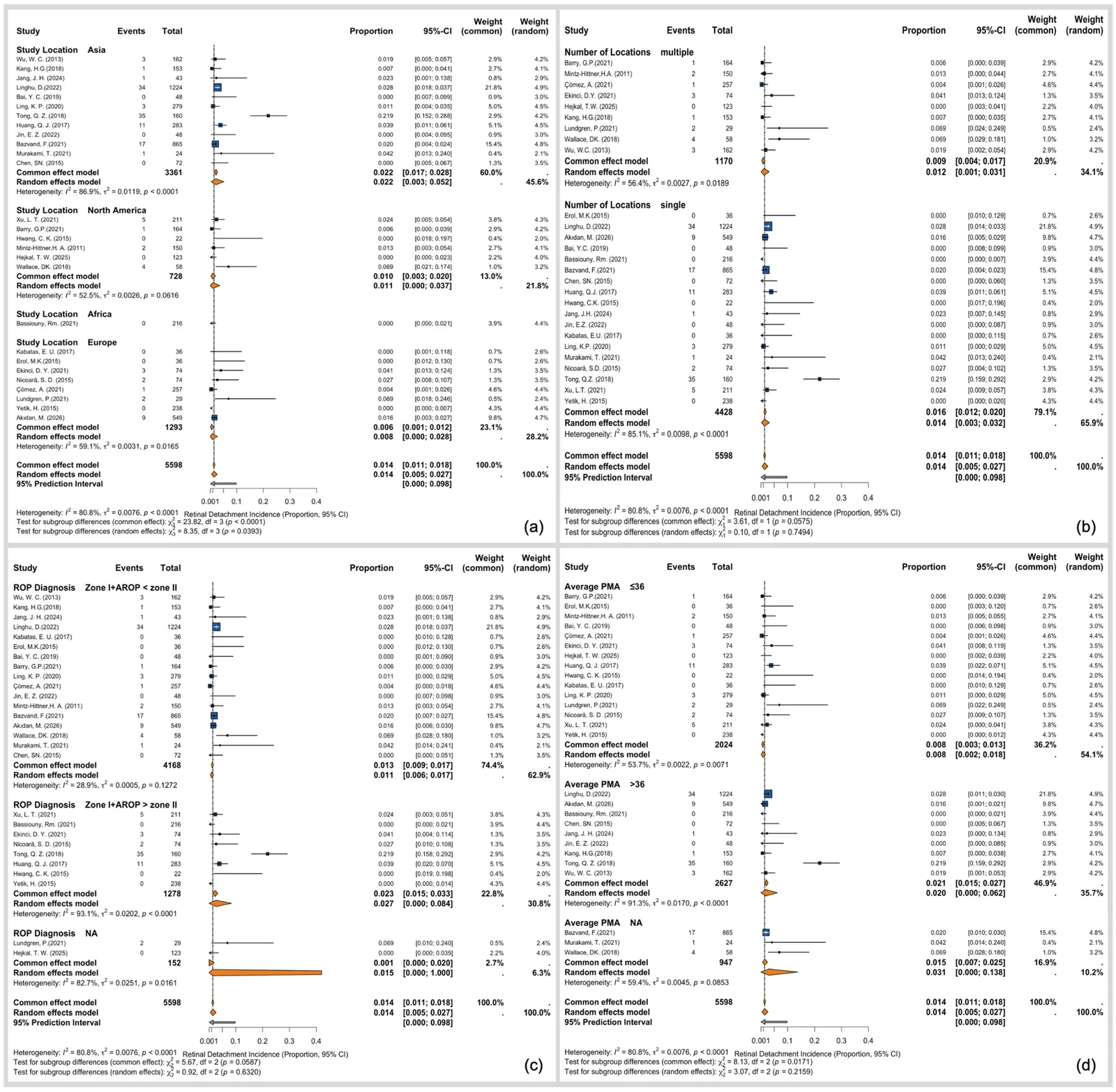

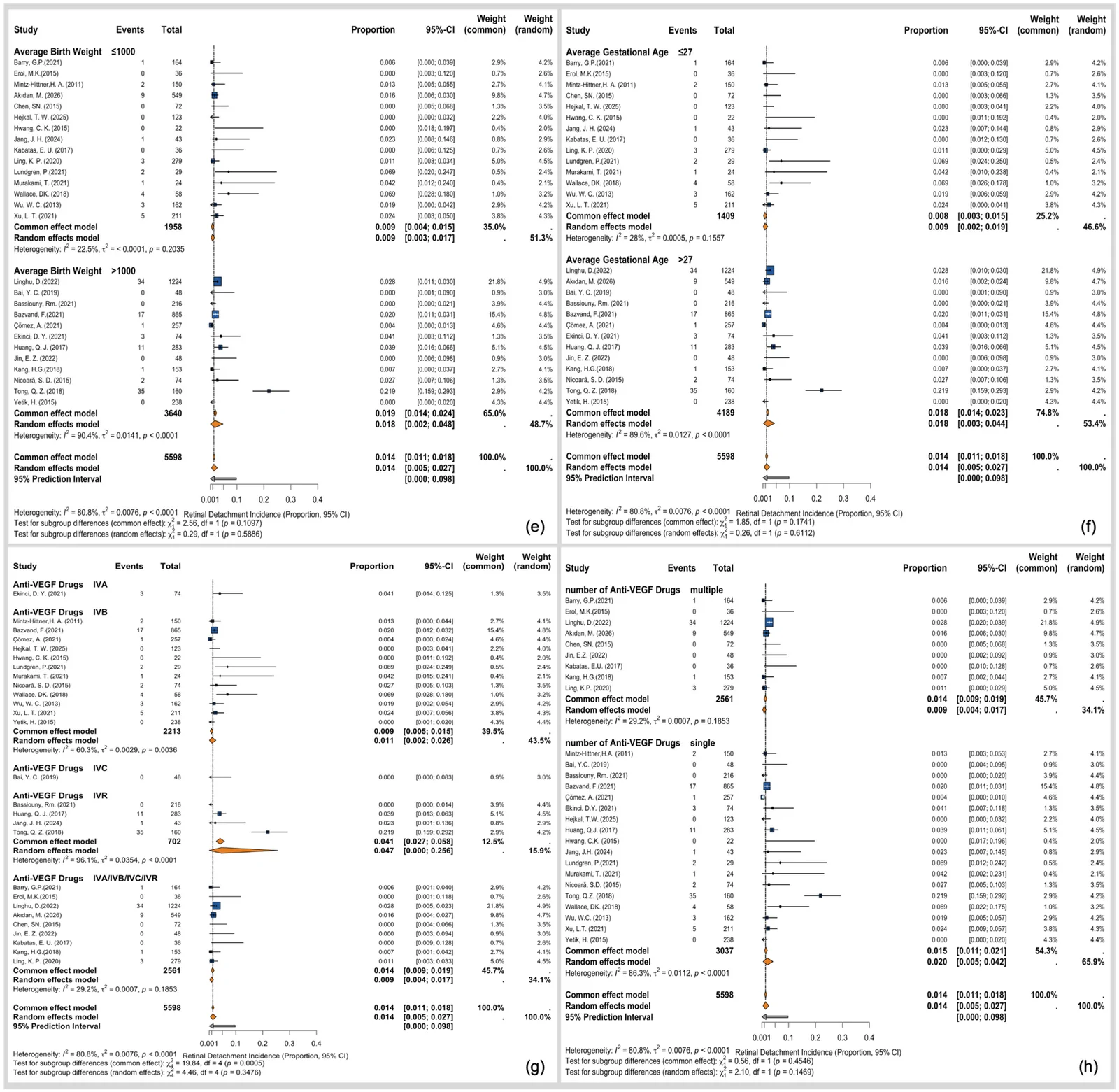
The results of subgroup analysis. (**a**) subgroup analysis of geographic location (Europe, Asia, North America, or Africa); (**b**) subgroup analysis of study design (single-center vs. multicenter); (**c**) subgroup analysis of predominant ROP zone (Zone I/A-ROP vs. Zone II); (**d**) subgroup analysis of PMA at treatment (≤36 vs. >36 weeks); (**e**) subgroup analysis of mean BW (≤1000 vs. >1000 g); (**f**) subgroup analysis of mean GA (≤27 vs. >27 weeks); (**g**) subgroup analysis of anti-VEGF drug strategy (IVA, IVB, IVC, or unspecified drug); (**h**) subgroup analysis of anti-VEGF drugs (single-drug vs. multidrug). Different colours are used to distinguish pooled incidence estimates of different subgroups.

Subsequently, in the subgroup analysis according to the main location of included studies, it was detected that the incidence of RD was highest in Asia 2.2% (95% CI 0.3–5.2%), followed by North America 1.1% (95% CI 0.0–3.7%), then Europe 0.8% (95% CI 0.0–2.8%) (Figure 4a). As for the number of locations, the incidence of RD was slightly higher for studies in a single center (1.4%, 95% CI 0.3–3.2%) than in multiple centers (1.2%, 95% CI 0.1–3.1%) (Figure 4b).

Based on our predefined criteria for subgroup analysis, ten studies were included. Two separate studies by Lundgren et al. [13] and Hejkal et al. [22] were excluded because they lacked the specific participants’ location diagnosis of ROP. In our analysis, when the number of patients with ROP (zone I) and AROP exceeded those with ROP (zone II), the risk difference for RD was 2.7% (95% CI 0.0–8.4%). Conversely, when cases of ROP (zone I) and AROP were less numerous than those in the ROP (zone II) group, the RD was slightly lower at 1.1% (95% CI 0.6–1.7%) (Figure 4c).

When the analysis was stratified by patient characteristics, GA, BW and PMA were found to be significant factors. The subgroup analysis based on PMA showed a significantly higher incidence of RD in patients with a PMA >36 weeks (2.0%, 95% CI 0.0–6.2%) than in those with a PMA ≤36 weeks (0.8%, 95% CI 0.2–1.8%) (Figure 4d). A slightly higher risk difference for RD was observed in patients with a BW of more than 1000 g (1.8%, 95% CI 0.2–4.8%) relative to those with a BW lower than 1000 g (0.9%, 95% CI 0.3–1.7%) (Figure 4e). This finding was similar in the analysis of GA, where neonates with a GA >27 weeks had a slightly higher RD (1.8%, 95% CI 0.3–4.4%) compared to those with a GA <27 weeks (0.9%, 95% CI 0.2–1.9%) (Figure 4f).

RD incidence varied across anti-VEGF drug types. Studies using ranibizumab showed a higher pooled reported RD incidence (4.7%, 95% CI, 0.0–25.6%) than those using bevacizumab (1.1%, 95% CI, 0.2–2.6%), then followed by unspecified drug strategy (0.9%, 95% CI 0.4–1.7%) (Figure 4g). In the subgroup analysis based on the number of anti-VEGF agents used, the incidence of RD was higher in the single-agent treatment group (2.0%, 95% CI 0.5–4.2%) than in the multiple-agent treatment group (0.9%, 95% CI 0.4–1.7%) (Figure 4h).

### 3.6. Publication Bias

**Figure 5:**
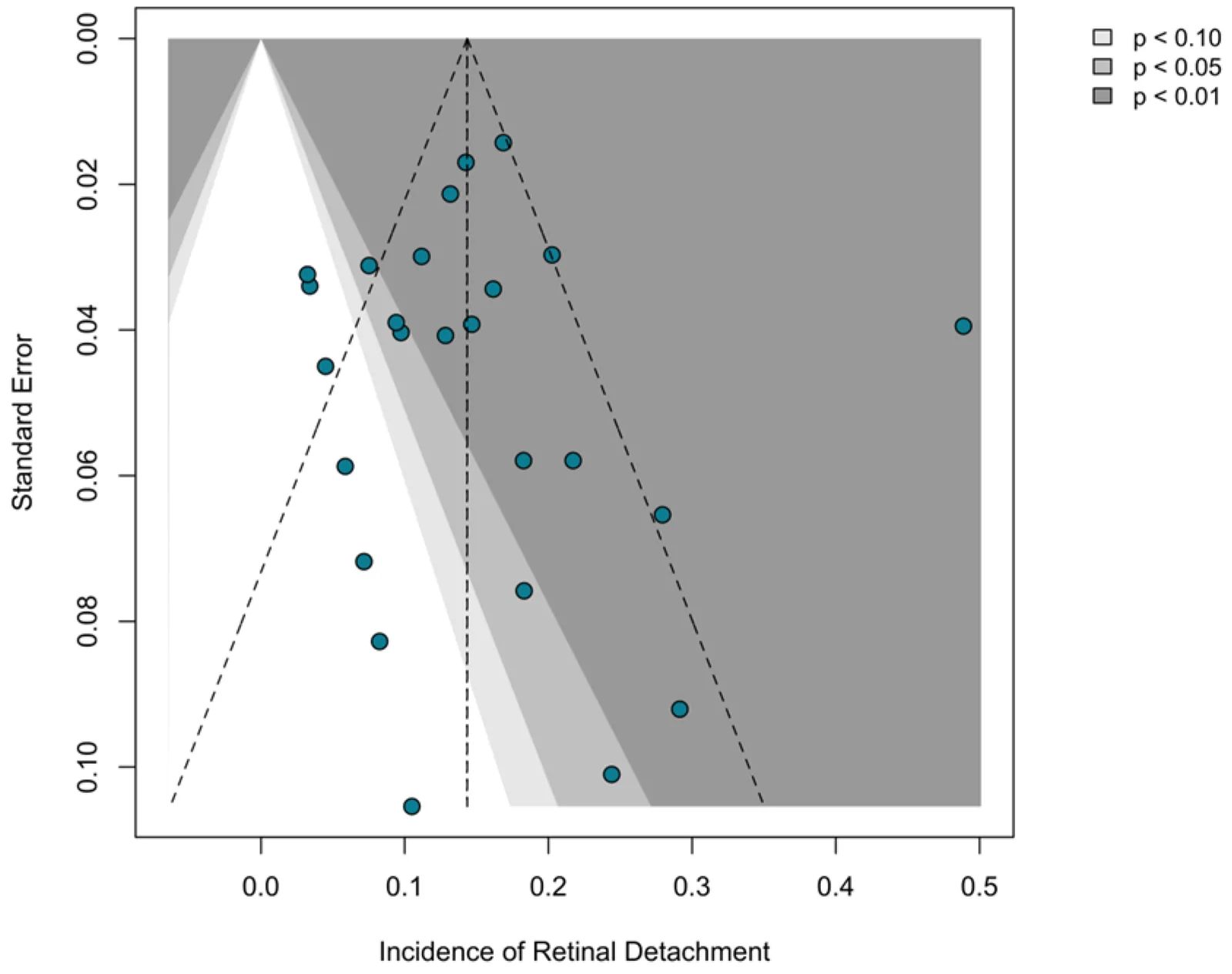
Funnel plot of the risk of publication bias for the prevalence of RD after anti-VEGF therapy in ROP. The sloped dashed lines and shaded regions denote the 90%, 95%, and 99% confidence limits for the funnel plot, used to assess potential publication bias.

The funnel plot showed an overall symmetrical distribution of included studies around the pooled effect size within the pseudo-95% confidence regions (Figure 5). Despite a single outlying point on the right side of the plot [33], no obvious asymmetry was detected, indicating no apparent publication bias. This was further supported by Egger’s and Begg’s tests, both of which did not indicate significant publication bias (Supplementary Material S1).

## 4. Discussion

This systematic review and meta-analysis, incorporating 27 published studies and 5598 treated eyes, provides the most comprehensive synthesis to date of reported RD incidence among infants with ROP treated primarily with anti-VEGF agents. The pooled incidence of reported RD was 1.4% (95% CI, 0.5–2.7%), with substantial heterogeneity across studies (*I*^2^ = 80.8%). Leave-one-out sensitivity analysis confirmed the robustness of the pooled estimate and identified the study by Tong et al. [33] as the principal source of heterogeneity; the exclusion of this single study reduced *I*^2^ to 56.1% while the pooled estimate shifted only modestly, to 1.0% (95% CI, 0.4–1.7%), suggesting that the heterogeneity was driven primarily by one outlying study rather than by fundamental inconsistency across the literature. The pooled estimate was consistent with previously reported rates from individual studies, which ranged from 0% in early-treatment subgroups to approximately 2.7–3.4% in higher-risk populations [11,12,13]. No single mechanism has been identified that fully accounts for why a subset of treated eyes progresses to RD despite otherwise successful disease regression. The existing literature, however, converges on a multifactorial explanation in which disease severity, treatment timing, post-injection fibrovascular dynamics, persistent retinal avascularity, and individual genetic susceptibility factors collectively shape the risk of this adverse outcome [11,42,43,44,45].

In our subgroup analysis, the severity of ROP was a critical determinant of reported RD incidence. Studies with a predominance of Zone I ROP or AROP yielded a substantially higher pooled RD estimate (2.7%) than those with a predominance of Zone II disease (1.1%). This gradient is consistent with the understanding that post-treatment RD is driven primarily by the underlying severity of ROP, as Zone I disease and AROP carry the highest intrinsic risk of unfavorable anatomical progression, irrespective of the treatment administered [46,47], and anti-VEGF agents are now recommended as the preferred primary treatment for precisely these severe forms [5,14,26]. The higher observed RD incidence in the Zone I/AROP subgroup therefore does not imply that anti-VEGF therapy is inappropriate for severe disease. On the contrary, current treatment guidelines explicitly recognize anti-VEGF agents as the preferred primary intervention for Zone I disease and AROP precisely because these eyes stand to benefit most from the rapid neovascular regression and the potential for continued peripheral vascularization that anti-VEGF therapy affords [48]. Zone I disease and AROP are characterized by profound retinal immaturity, a massive VEGF load, and a greater propensity for fibrovascular proliferation, all of which create a structural substrate that is inherently susceptible to tractional detachment, irrespective of the treatment modality employed [49,50]. Within this framework, the RD events captured in our analysis are best understood as the residual, imperfect anatomical outcomes that occur at the most severe end of the ROP spectrum despite effective treatment, rather than as evidence of anti-VEGF-induced disease worsening. This interpretation underscores the importance of post-treatment surveillance and the need for prolonged, risk-stratified follow-up in eyes with Zone I or AROP, where the competing forces of disease regression and fibrovascular contraction demand particularly vigilant monitoring [43].

However, the findings for GA, BW, and PMA at treatment initially appear counterintuitive. Higher reported RD incidence was associated with studies reporting a mean GA exceeding 27 weeks (1.8% vs. 0.9%), mean BW exceeding 1000 g (1.8% vs. 0.9%), and mean PMA at treatment greater than 36 weeks (2.0% vs. 0.8%). Although these results seemingly contradict the well-established epidemiological pattern that lower GA and BW confer greater ROP severity [41], they align with the recognition that more mature preterm infants remain at risk for severe, treatment-requiring ROP [51]. This also aligns with Barry et al.’s finding that RD after anti-VEGF therapy was more frequent among infants treated at a PMA greater than 36 weeks [11]. Because no research has directly examined the relationship between GA, BW, or PMA at treatment and anatomical outcomes after anti-VEGF therapy, the subgroup patterns observed in this analysis must be interpreted through indirect evidence and biological considerations. First, these subgroups were defined by study-level means, and the within-study distributions of GA, BW, and PMA likely overlapped substantially. A study-level subgroup analysis cannot recover the patient-level gradient of risk and is susceptible to ecological bias [52]. Second, infants with higher GA and BW who nonetheless develop treatment-requiring ROP may represent a biologically distinct subgroup [53]. Reaching the treatment threshold despite being less premature suggests a particularly aggressive disease course. In line with this interpretation, Yonekawa et al. proposed that the upregulation of TGF-β as preterm infants approach term creates a profibrotic milieu, promoting fibrovascular membrane contraction and tractional detachment [54]. This mechanism may partially explain why eyes treated at a later PMA, or those in infants with higher GA, are more susceptible to post-treatment RD despite their apparently greater somatic maturity. Third, PMA at treatment may be a more direct determinant of RD risk than GA or BW. Infants treated at a later PMA may have experienced a longer interval of active, untreated neovascularization, allowing more extensive fibrovascular membranes to form. Because preexisting fibrovascular membranes are known to undergo acute contraction after VEGF suppression [55], this interpretation is consistent with Barry et al.’s observation that RD incidence rose with increasing PMA at treatment [11], and with Linghu et al.’s finding that earlier disease control was associated with better anatomical outcomes [26]. Clinically, these findings caution that treatment-requiring ROP in relatively large or old preterm infants should not be underestimated. Furthermore, timely intervention before extensive fibrovascular proliferation occurs is critical, regardless of GA or BW.

Although current ROP management worldwide follows common international guidelines [48], regional implementation varies owing to differences in screening infrastructure, treatment access, and follow-up adherence [56,57,58,59]. These disparities are reflected in the geographic distribution of reported RD incidence in our analysis. The higher RD incidence observed in studies from Asia (2.2%) relative to Europe (0.8%) and North America (1.1%) warrants careful interpretation. Asia currently carries the largest burden of ROP worldwide, which aligns with the “third epidemic” driven by rapid improvements in neonatal survival coupled with variable screening infrastructure and treatment access [60]. The higher reported RD rate in Asian studies may reflect later presentation, a higher proportion of AROP cases, or differences in treatment protocols and follow-up adherence [61]. Conversely, the lower rates in North American and European studies likely reflect more mature healthcare systems with earlier detection and standardized treatment protocols [62,63]. Additionally, the single African study yielded a very low RD estimate, which likely reflects a small sample size and limited generalizability rather than true epidemiological differences [64,65]. A similar pattern emerged when comparing single-center and multicenter studies. Single-center studies reported a higher RD incidence than multicenter studies (1.4% vs. 1.2%), a finding consistent with referral bias [66]. Single centers that publish research often function as regional referral hubs managing a disproportionate share of complex, treatment-challenging cases [67].

The subgroup analysis by anti-VEGF agent revealed the highest pooled RD incidence among studies using ranibizumab (4.7%), followed by bevacizumab (1.1%) and the unspecified drug subgroup (0.9%). However, the ranibizumab estimate yielded a notably wide confidence interval (0.0–24.5%) and warrants cautious interpretation. This finding is unlikely to reflect a true pharmacological difference between ranibizumab and bevacizumab regarding RD risk; rather, it suggests confounding by indication. Ranibizumab is often preferentially selected for Zone I disease and AROP in clinical practice, particularly in Asian settings, where several included studies originated [33,35]. Therefore, the higher RD rate in the ranibizumab subgroup is more plausibly attributable to the greater baseline severity of the treated eyes than to any inherent property of the drug [11,68,69]. Similarly, the finding that the single-agent subgroup exhibited a higher RD incidence than the multiple-agent subgroup (2.0% vs. 0.9%) should not be interpreted as evidence that multidrug therapy confers a protective effect. Instead, this pattern likely reflects differences in retreatment protocols, follow-up intensity, and disease severity distributions across the studies within each subgroup [15,70]. These drug-related subgroup findings underscore that, within the current ROP literature, treatment selection is inherently linked to disease severity, and unadjusted comparisons inevitably conflate disease-driven risk with treatment-attributed risk [70,71]. Furthermore, the potential influence of genetic factors on patient sensitivity to anti-VEGF therapy cannot be excluded [45]. Future prospective studies should therefore adopt designs capable of isolating these factors to provide more actionable evidence for clinical decision-making. Such designs must incorporate randomization stratified by ROP zone and severity, standardized retreatment criteria, and prespecified subgroup analyses.

In this meta-analysis, a central question raised by subgroup analysis is whether the RD events captured in all included studies are attributable to anti-VEGF therapy itself or primarily reflect the natural history of severe ROP. Although the available data do not permit a definitive answer, the temporal relationship between injection and RD, when available, offers a useful framework for distinguishing among potential mechanisms [43]. In an international multicenter study of eyes that developed RD after anti-VEGF treatment, Yonekawa et al. reported that progression to RD occurred at a mean of 70 days (approximately 10 weeks) after injection, with 11% of detachments occurring within 1 week and 49% within 4 weeks. Notably, the time to detachment was negatively correlated with PMA at injection: younger infants exhibited a longer latency before detachment, whereas older infants detached more rapidly [72]. This temporal distribution is consistent with the series reported by Kondo et al., in which the median interval from injection to RD was approximately 10 weeks [9], and suggests that the majority of post-injection RD events reflect a gradual fibrovascular contracture process rather than an abrupt overnight event.

The classic “crunch” phenomenon, defined as the acute contraction of preexisting fibrovascular membranes within a few days to two weeks of VEGF suppression, has been described in multiple reports [55,73] and likely accounts for a small subset of early post-injection detachments. Three distinct anatomical configurations of crunch-related RD have been characterized, namely conventional progression, very posterior detachment with prepapillary contraction, and relatively peripheral detachment with tight circumferential tractional vectors. It has been hypothesized that anti-VEGF agents may induce fibrosis and contraction of immature prepapillary vascular precursor cells in the prepapillary configuration, and of flat neovascularization in the circumferential configuration. The conventional configuration was associated with successful anatomical repair in all cases, whereas the prepapillary and circumferential configurations each had an anatomical success rate of approximately two-thirds, underscoring the surgical challenge posed by these atypical detachments [54]. A rare but distinct early mechanism is exudative RD secondary to acute choroidal ischemia following anti-VEGF injection, which may present within hours and resolve with corticosteroid and laser therapy [74]. Furthermore, isolated reports of rhegmatogenous RD associated with distinct retinal holes following anti-VEGF therapy may suggest a rare iatrogenic etiology related to needle trauma during the injection procedure [40].

At intermediate latency, Hu et al. described reactivation-driven RD occurring 14 to 34 weeks (median approximately 20 weeks) after bevacizumab monotherapy [75], a pattern that may reflect incomplete disease suppression and the subsequent contraction of residual fibrovascular tissue. At the late end of the spectrum, RD has been reported 1 year [76], 2.5 years [77], and even 11 years [78] after anti-VEGF treatment, events that are most plausibly attributed to persistent retinal avascularity and chronic vitreoretinal traction rather than to any acute pharmacological effect [44,79]. Supporting this interpretation, an animal model of ROP demonstrated that anti-VEGF antibody treatment led to late atypical intravitreal neovascularization and the reactivation of angiogenic pathways [80], providing a biological rationale for the extended period of risk. These time-stratified patterns highlight a critical gap in the current literature, as the timing and anatomical subtype (tractional, rhegmatogenous, exudative or combined) of RD after anti-VEGF treatment are rarely reported. Without such data, distinguishing treatment-associated events from the natural progression of ROP remains challenging, and pooling etiologically distinct events may obscure important differences in pathophysiology and management. Future studies should systematically report the timing, subtype, and clinical characteristics of RD to clarify potential mechanisms, identify high-risk periods, and inform post-treatment surveillance.

Based on the above considerations, rather than viewing anti-VEGF agents as the sole independent cause of RD, we consider these events to be multifactorial. The RD events reported in the literature appear to be predominantly driven by the severity of the underlying disease and the consequent vulnerability of the retinal structure that provides the anatomical substrate of fibrovascular membranes; they are frequently catalyzed by acute or subacute post-injection fibrovascular contraction, with a very small number attributable to procedural trauma.

Several limitations of the current evidence base must be acknowledged. First, the included studies were almost exclusively retrospective, and treatment allocation was non-randomized, introducing potential confounding by indication that cannot be fully adjusted for in a study-level meta-analysis. The limited availability of RCTs further reduces the certainty of the evidence and highlights the need for adequately powered randomized studies to confirm these findings. Second, outcomes were reported per eye rather than per infant in the majority of included studies; the non-independence of fellow eyes may underestimate the true variance of the pooled estimate. Third, the therapeutic regimens varied considerably across studies in terms of drug type, dose, number of injections, retreatment criteria, and the use of supplemental laser, all of which may influence RD risk. Fourth, as discussed above, the pervasive lack of granular data regarding RD timing and anatomical subtype precluded us from performing time-stratified subgroup analyses to further uncouple drug-induced “crunch” from the natural progression of the disease. Furthermore, the included studies rarely reported post-RD visual outcomes, limiting our ability to assess the long-term functional consequences of these events. Fifth, follow-up duration varied substantially (from 8 weeks to over 2 years), and studies with shorter observation periods may underestimate the true cumulative incidence of RD, particularly late events.

## 5. Conclusions

In this systematic review and meta-analysis of 27 studies involving 5598 treated eyes, the pooled incidence of reported RD following primary anti-VEGF therapy for ROP was 1.4% (95% CI, 0.5–2.7%), with substantial heterogeneity across studies. Incidence rates varied significantly by baseline disease severity, peaking in cohorts characterized by Zone I ROP or AROP, as well as in Asian and single-center cohorts. These trends strongly suggest confounding by indication rather than direct drug-related causation, as anti-VEGF therapy is preferentially used in eyes at the highest baseline risk for RD. Consequently, post-treatment RD should be interpreted as a residual adverse anatomical outcome inherent to severe ROP, rather than evidence of therapy-induced disease exacerbation. Clinically, these findings support a risk-stratified surveillance strategy: infants with Zone I ROP, AROP, or persistent/recurrent disease warrant prolonged multidisciplinary follow-up between pediatric ophthalmologists and neonatologists to optimize treatment timing and systemic stability. Importantly, the risk of RD should not deter clinicians from using anti-VEGF therapy in severe ROP when laser treatment is infeasible or undesirable. To address existing evidentiary limitations, future prospective studies must adopt standardized RD definitions (detailing specific post-treatment timing, anatomical subtype, and severity staging), incorporate severity-adjusted comparisons, and ensure sufficiently extended follow-up to capture late events.

## Figures and Tables

**Table 2:** Summary of included study characteristics, stratified by subgroup.

Study	Therapied Eyes	RD Eyes After Treatment	Location	Number of Locations	Average GA, Weeks	Average BW, Gram	Average PMA, Weeks	Number of ROP (Zone I) & AROP vs. ROP (Zone II)	Anti-VEGF Drugs	Number of Drugs
Wu, W.C. 2013 [23]	162	3	Asia	multiple	≤27	≤1000	>36	Zone I + AROP < zone II	IVB	single
Kang, H.G. 2018 [21]	153	1	Asia	multiple	>27	>1000	>36	Zone I + AROP < zone II	multiple	multiple
Jang, J.H. 2024 [24]	43	1	Asia	single	≤27	≤1000	>36	Zone I + AROP < zone II	IVR	single
Xu, L.T. 2021 [20]	211	5	N. Am.	single	≤27	≤1000	≤36	Zone I + AROP > zone II	IVB	single
Bassiouny, Rm. 2021 [25]	216	0	Africa	single	>27	>1000	>36	Zone I + AROP > zone II	IVR	single
Linghu, D. 2022 [26]	1224	34	Asia	single	>27	>1000	>36	Zone I + AROP < zone II	multiple	multiple
Kabatas, E.U. 2017 [27]	36	0	Europe	single	≤27	≤1000	≤36	Zone I + AROP < zone II	multiple	multiple
Erol, M.K. 2015 [28]	36	0	Europe	single	≤27	≤1000	≤36	Zone I + AROP < zone II	multiple	multiple
Ekinci, D.Y. 2021 [29]	74	3	Europe	multiple	>27	>1000	≤36	Zone I + AROP > zone II	IVA	single
Bai, Y.C. 2019 [30]	48	0	Asia	single	>27	>1000	≤36	Zone I + AROP < zone II	IVC	single
Barry, G.P. 2021 [11]	164	1	N. Am.	multiple	≤27	≤1000	≤36	Zone I + AROP < zone II	multiple	multiple
Ling, K.P. 2020 [31]	279	3	Asia	single	≤27	≤1000	≤36	Zone I + AROP < zone II	multiple	multiple
Nicoară, S.D. 2015 [32]	74	2	Europe	single	>27	>1000	≤36	Zone I + AROP > zone II	IVB	single
Tong, Q.Z. 2018 [33]	160	35	Asia	single	>27	>1000	>36	Zone I + AROP > zone II	IVR	single
Çömez, A. 2021 [34]	257	1	Europe	multiple	>27	>1000	≤36	Zone I + AROP < zone II	IVB	single
Huang, Q.J. 2017 [35]	283	11	Asia	single	>27	>1000	≤36	Zone I + AROP > zone II	IVR	single
Hwang, C.K. 2015 [36]	22	0	N. Am.	single	≤27	≤1000	≤36	Zone I + AROP > zone II	IVB	single
Jin, E.Z. 2022 [37]	48	0	Asia	single	>27	>1000	>36	Zone I + AROP < zone II	multiple	multiple
Mintz-Hittner, H.A. 2011 [12]	150	2	N. Am.	multiple	≤27	≤1000	≤36	Zone I + AROP < zone II	IVB	single
Lundgren, P. 2021 [13]	29	2	Europe	multiple	≤27	≤1000	≤36	NA	IVB	single
Bazvand, F. 2021 [19]	865	17	Asia	single	>27	>1000	NA	Zone I + AROP < zone II	IVB	single
Hejkal, T.W. 2025 [22]	123	0	N. Am.	multiple	≤27	≤1000	≤36	NA	IVB	single
Yetik, H. 2015 [38]	238	0	Europe	single	>27	>1000	≤36	Zone I + AROP > zone II	IVB	single
Akıdan, M. 2026 [10]	549	9	Europe	single	>27	≤1000	>36	Zone I + AROP < zone II	multiple	multiple
Wallace, D.K. 2018 [39]	58	4	N. Am.	multiple	≤27	≤1000	NA	Zone I + AROP < zone II	IVB	single
Murakami, T. 2021 [40]	24	1	Asia	single	≤27	≤1000	NA	Zone I + AROP < zone II	IVB	single
Chen, S.N. 2015 [41]	72	0	Asia	single	≤27	≤1000	>36	Zone I + AROP < zone II	multiple	multiple

## Data Availability

The data supporting the findings of this study are not publicly available but are available from the corresponding author upon reasonable request.

## Supplementary Materials

The following supporting information can be downloaded at: https://www.mdpi.com/article/10.3390/jcm15197598/s1. Supplementary Materials S1: Section SA: Record of database search strategies. Section SB: References of Included studies. Supplementary Figure S1: After excluding specific studies, the forest plot of the effect sizes (ES) with 95% confidence intervals in individual studies and overall pooled effect and Funnel plot of the risk of publication bias. Supplementary Table S1: The results of Egger’s and Begg’s test for the included studies. Supplementary Table S2: Anti-VEGF treatment details for ROP patients in 27 included studies. Supplementary Table S3: Clinical outcomes after treatment for ROP patients in 27 included studies. Supplementary Materials S2: PRISMA 2020 Checklist.

## Abbreviations

The following abbreviations are used in this manuscript:

ROP	Retinopathy of Prematurity
VEGF	Vascular Endothelial Growth Factor
AROP	Aggressive Retinopathy of Prematurity
RD	Retinal Detachment
GA	Gestational Age
BW	Birth Weight
PMA	Postmenstrual Age
TGF-β	Transforming Growth Factor—Beta
RCT	Randomized Controlled Trial
