# Supplementary material for: Reported Retinal Detachment in Anti-VEGF-Treated Retinopathy of Prematurity: A Systematic Review and Meta-Analysis
Source: J Clin Med. 2026 Sep 30;15(19):7598. doi: 10.3390/jcm15197598 (PMC13666268; doi:10.3390/jcm15197598)
Supplement: Supplementary file 1 [file jcm-15-07598-s001.zip › jcm-4519634-Supplementary Materials S1.pdf]

Supplementary Materials S1

Section SA. Record of database search strategies

EMBASE search strategy:
#1 'retinopathy of prematurity'/exp OR 'retinopathy of prematurity':ti,ab OR ROP:ti,ab
#2 'vascular endothelial growth factor inhibitor'/exp OR 'vascular endothelial growth factor a'/exp OR 'anti-vegf':ti,ab OR 'vegf inhibitor*':ti,ab OR bevacizumab:ti,ab OR ranibizumab:ti,ab OR aflibercept:ti,ab OR conbercept:ti,ab
#3 'retinal detachment'/exp OR 'retinal detachment':ti,ab OR 'tractional retinal detachment':ti,ab OR RD:ti,ab OR TRD:ti,ab

PubMed search strategy:
#1 ("Retinopathy of Prematurity"[MeSH Terms] OR "retinopathy of prematurity"[Title/Abstract] OR ROP[Title/Abstract])
#2 ("Vascular Endothelial Growth Factor A"[MeSH Terms] OR "VEGF"[Title/Abstract] OR "anti-VEGF"[Title/Abstract] OR "VEGF inhibitor*"[Title/Abstract] OR bevacizumab[Title/Abstract] OR ranibizumab[Title/Abstract] OR aflibercept[Title/Abstract] OR conbercept[Title/Abstract] OR pegaptanib[Title/Abstract])
#3 ("Retinal Detachment"[MeSH Terms] OR "retinal detachment"[Title/Abstract] OR RD[Title/Abstract] OR "tractional retinal detachment"[Title/Abstract] OR TRD[Title/Abstract] OR "exudative retinal detachment"[Title/Abstract] OR ERD[Title/Abstract] OR "recurrent retinal detachment"[Title/Abstract])

Cochrane Library search strategy:
#1 (MeSH descriptor: [Retinopathy of Prematurity] explode all trees OR "retinopathy of prematurity":ti,ab,kw OR ROP:ti,ab,kw)

#2 (MeSH descriptor: [Vascular Endothelial Growth Factor A] explode all trees OR MeSH descriptor: [Angiogenesis Inhibitors] explode all trees OR "vascular endothelial growth factor":ti,ab,kw OR VEGF:ti,ab,kw OR "anti-VEGF":ti,ab,kw OR "VEGF inhibitor*":ti,ab,kw OR bevacizumab:ti,ab,kw OR ranibizumab:ti,ab,kw OR aflibercept:ti,ab,kw OR conbercept:ti,ab,kw OR pegaptanib:ti,ab,kw)
#3 (MeSH descriptor: [Retinal Detachment] explode all trees OR "retinal detachment":ti,ab,kw OR "tractional retinal detachment":ti,ab,kw OR "exudative retinal detachment":ti,ab,kw OR "recurrent retinal detachment":ti,ab,kw OR RD:ti,ab,kw OR TRD:ti,ab,kw OR ERD:ti,ab,kw).

Web of Science search strategy:
#1 TS=("Retinopathy of Prematurity" OR "retinopathy of prematurity" OR ROP)
#2 TS=("Vascular Endothelial Growth Factor" OR VEGF OR anti-VEGF OR "VEGF inhibitor*" OR bevacizumab OR ranibizumab OR aflibercept OR conbercept OR pegaptanib)
#3 TS=("Retinal Detachment" OR RD OR "tractional retinal detachment" OR TRD OR "exudative retinal detachment" OR ERD OR "recurrent retinal detachment").

Section SB. References of Included studies

1. Barry G, Yu Y, Ying G, et al. Retinal detachment after treatment of retinopathy of prematurity with laser versus intravitreal anti-vascular endothelial growth factor. *Ophthalmology*. 2021;128(8):1188-1196.

2. Erol M, Coban D, Sari E, et al. Comparison of intravitreal ranibizumab and bevacizumab treatment for retinopathy of prematurity. *Arq Bras Oftalmol*. 2015;78(6):340-343.

3. Murakami T, Sugiura Y, Okamoto F, et al. Comparison of 5-year safety and efficacy of laser photocoagulation and intravitreal bevacizumab injection in retinopathy of prematurity. *Graefes Arch Clin Exp Ophthalmol*. 2021;259(9):2849-2855.

4. Lundgren P, Jacobson L, Hård AL, et al. High rate and large intercentre variability in retreatment of retinopathy of prematurity in infants born <24 gestational weeks. *BMJ Open Ophthalmol*. 2021;6(1):e000695.

5. Wallace DK, Dean TW, Hartnett ME, et al. A dosing study of bevacizumab for retinopathy of prematurity. *Ophthalmology*. 2018;125(12):1961-1966.

6. Çömez A, Karaküçük Y, Özmen M, Çelemler P, Saygili O. The results of intravitreal bevacizumab monotherapy for treating aggressive posterior retinopathy of prematurity and type 1 retinopathy of prematurity. *EYE*. 2021;35(12):3302-3310.

7. Wu WC, Kuo HK, Yeh PT, Yang CM, Lai CC, Chen SN. An updated study of the use of bevacizumab in the treatment of patients with prethreshold retinopathy of prematurity in taiwan. *Am J Ophthalmol*. 2013;155(1):150-158.

8. Kang HG, Choi EY, Byeon SH, et al. Anti-vascular endothelial growth factor treatment of retinopathy of prematurity: efficacy, safety, and anatomical outcomes. *Korean j ophthalmol : KJO*. 2018;32(6):451-458.

9. Jang J. Characteristics of retinal vascularization in reactivated retinopathy of prematurity requiring treatment and clinical outcome after reinjection of ranibizumab. *Sci Rep*. 2024;14(1):15647.

10. Xu L, Levine D, Hutchinson A, Rao P, Hubbard G. Clinical features and outcomes of infants with retinopathy of prematurity who fail antivascular endothelial growth factor therapy. *RETINA-THE J OF RETIN AND VITR DIS*. 2021;41(11):2269-2277.

11. Bassiouny R, Gaafar W, El Nokrashy A, et al. Clinical outcome following reinjection of ranibizumab for reactivation of retinopathy of prematurity. *EYE*. 2022;36(11):2137-2143.

12. Linghu D, Cheng Y, Zhu X, et al. Comparison of intravitreal anti-VEGF agents with laser photocoagulation for retinopathy of prematurity of 1,627 eyes in China. *Front Med*. 2022;9((Linghu D.; Cheng Y.; Zhu X.; Deng X.; Yin H.; Jiang Y.; Zhao M.; Li X.; Liang J., drliangjianhong@126.com) Department of Ophthalmology, Peking University People's Hospital, Eye Diseases and Optometry Institute, Beijing, China):911095.

13. Kabatas E, Kurtul B, Özer P, Kabatas N. Comparison of intravitreal bevacizumab, intravitreal ranibizumab and laser photocoagulation for treatment of type 1 retinopathy of prematurity in turkish preterm children. *Curr Eye Res*. 2017;42(7):1054-1058.

14. Ekinci D, Vural A. Comparison of two different doses of intravitreal aflibercept in the treatment of retinopathy of prematurity. *J AAPOS*. 2021;25(2):93.e1-93.e5.

15. Bai Y, Nie H, Wei S, et al. Efficacy of intravitreal conbercept injection in the treatment of retinopathy of prematurity. *Br J Ophthalmol*. 2019;103(4):494-498.

16. Hejkal TW, Sinha S, Rishi P, Jain SF, Rychwalski PJ. Low-dose bevacizumab 0.03 mg for treatment of type 1 retinopathy of prematurity. *Ophthalmol, Retina*. 2025;9(8):807-811.

17. Hwang C, Hubbard G, Hutchinson A, Lambert S. Outcomes after intravitreal bevacizumab versus laser photocoagulation for retinopathy of prematurity a 5-year retrospective analysis. *Ophthalmology*. 2015;122(5):1008-1015.

18. Nicoara S, Nascutzy C, Cristian C, et al. Outcomes and prognostic factors of intravitreal bevacizumab monotherapy in zone I stage 3+and aggressive posterior retinopathy of prematurity. *J OF OPHTHALMOL*. 2015;2015:102582.

19. Tong Q, Yin H, Zhao M, Li X, Yu W. Outcomes and prognostic factors for aggressive posterior retinopathy of prematurity following initial treatment with intravitreal ranibizumab. *BMC Ophthalmol*. 2018;18(1):150.

20. Huang Q, Zhang Q, Fei P, et al. Ranibizumab injection as primary treatment in patients with retinopathy of prematurity anatomic outcomes and influencing factors. *Ophthalmology*. 2017;124(8):1156-1164.

21. Ling K, Liao P, Wang N, et al. Rates and risk factors for recurrence of retinopathy of prematurity after laser or intravitreal anti-vascular endothelial growth factor monotherapy. *RETINA-THE J OF RETIN AND VITR DIS*. 2020;40(9):1793-1803.

22. Jin E, Yin H, Li X, Zhao M. Short-term outcomes after intravitreal injections of conbercept versus ranibizumab for the treatment of retinopathy of prematurity. *RETINA-THE J OF RETIN AND VITR DIS*. 2018;38(8):1595-1604.

23. Yetik H, Gunay M, Sirop S, Salihoglu Z. Intravitreal bevacizumab monotherapy for type-1 prethreshold, threshold, and aggressive posterior retinopathy of prematurity — 27 month follow-up results from Turkey. *Graefes Arch Clin Exp Ophthalmol*. 2015;253(10):1677-1683.

24. Akıdan M, Ünal AC, Erol MK. Evaluation of regression, reactivation, retreatment and retinal detachment after injections of three different anti-VEGF drugs for retinopathy of prematurity. *Sci Rep*. Published online May 16, 2026.

25. Bazvand F, Riazi-Esfahani H, Mirshahi A, et al. Ocular complications following intravitreal bevacizumab injection for retinopathy of prematurity and assessment of risk factors. *Int J Retina Vitr*. 2021;7(1):5-12.

26. Chen SN, Lian I, Hwang YC, et al. Intravitreal anti-vascular endothelial growth factor treatment for retinopathy of prematurity: comparison between ranibizumab and bevacizumab. Retina. 2015;35(4):667-674.

27. Mintz-Hittner HA, Kennedy KA, Chuang AZ. Efficacy of intravitreal bevacizumab for stage 3+ retinopathy of prematurity. N Engl J Med. 2011;364(7):603-615.

Supplementary Figure S1. After excluding specific studies, the forest plot of the effect sizes (ES) with 95% confidence intervals in individual studies and overall pooled effect and Funnel plot of the risk of publication bias.

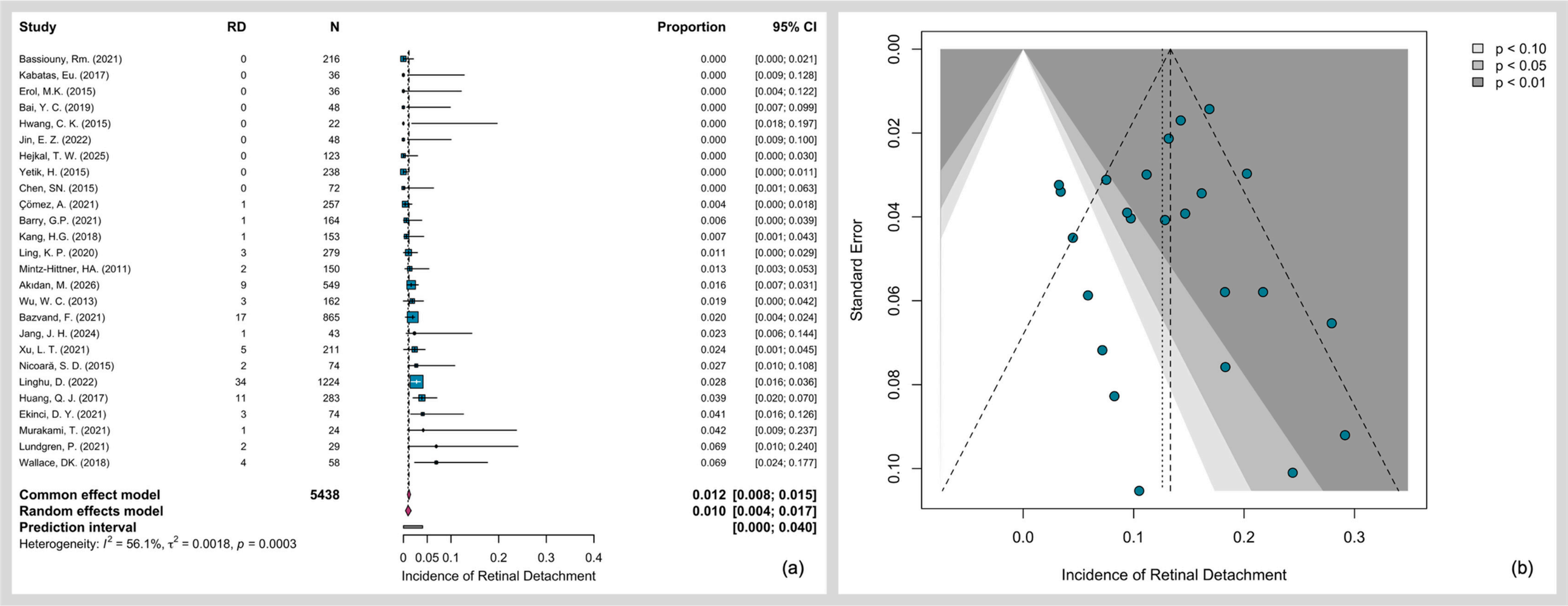

Supplementary Table S1. The results of Egger's and Begg's test for the included studies.

	Egger's test	Begg's test
**Test Result**	t = -0.09, df = 25	z = 0.79,
	p-value = 0.9304	p-value = 0.4278
**Bias estimate (SE)**	-0.0793 (0.8984)	38.0000 (47.9168)
	τ^2= 5.4252	

Supplementary Table S2. Anti-VEGF treatment details for ROP patients in 27 included studies.

First Author	Publication Year	Number of Therapied Eyes	Anti-VEGF Drugs (n, eyes)	Route of Administration	Dose of anti-VEGF Drugs	Salvage Treatment	Follow-up Duration*
Wu, W.C.	2013	162	Bevacizumab (162)	single injection	0.625mg/0.025mL	NA	13.7 ± 5.8 months
Kang, H.G.	2018	153	Bevacizumab (101)<br>Ranibizumab (52)	single injection	Bevacizumab: 0.625mg/0.025 mL<br>Ranibizumab: 0.2mg/0.02 mL	NA	25.1 ± 18.5 months
Jang, J.H.	2024	43	Ranibizumab (43)	single injection	0.2 mg/0.02 mL	anti-VEGF injection	70 ± 3 weeks
Xu, L.T.	2021	211	Bevacizumab (211)	single injection	0.031 - 0.625 mg<br>(multiple doses)	anti-VEGF injection / laser therapy / vitrectomy	2 years (Median)
Bassiouny, Rm.	2021	216	Ranibizumab (216)	single injection	0.25 mg/0.025 mL	anti-VEGF injection	at least 6 months after initial IVR injection
Linghu, D.	2022	1224	Bevacizumab (25)<br>Ranibizumab (916)<br>Conbercept (283)	single injection	Bevacizumab: 0.625 mg/0.025 mL<br>Ranibizumab: 0.25 mg/0.025 mL<br>Conbercept: 0.25 mg/0.025 mL	anti-VEGF injection / laser therapy	at least 6 months.
Kabatas, E.U.	2017	36	Bevacizumab (24)<br>Ranibizumab (12)	single injection	Bevacizumab: 0.625 mg/0.025 mL<br>Ranibizumab: 0.25 mg/0.025 mL	laser therapy	18 months
Erol, M.K.	2015	36	Bevacizumab (21)<br>Ranibizumab (15)	single injection	Bevacizumab: 0.625mg<br>Ranibizumab: 0.25mg	laser therapy	20 ± 4.5 months
Ekinci, D.Y.	2021	74	Aflibercept (74)	single injection	low dose: 0.4 mg/0.01 mL<br>standard dose: 1 mg/0.025 mL	low dose: laser therapy<br>standard dose: anti-VEGF injection / laser therapy	low dose: 34.1 ± 5.5 weeks<br>standard dose: 34.3 ± 7.5 weeks

Bai, Y.C.	2019	48	Conbercept (48)	single injection	0.25 mg/0.025 mL	anti-VEGF injection / laser therapy	31.0 ± 4.7 weeks
Barry, G.P.	2021	164	Bevacizumab (147)<br>Ranibizumab (17)	single injection	NA	NA	8 weeks after initial treatment
Ling, K.P.	2020	279	Bevacizumab (231)<br>Ranibizumab (48)	single injection	Bevacizumab: 0.625 mg/0.025 mL<br>Ranibizumab: 0.25 mg/0.025 mL	anti-VEGF injection / laser therapy	197.3 ± 110 weeks
Nicoară, S.D.	2015	74	Bevacizumab (74)	single injection	0.625 mg/0.025 mL	unclear，but no repeated anti-VEGF injections	60 weeks after injection
Tong, Q.Z.	2018	160	Ranibizumab (160)	single injection	0.3 mg/0.03 mL	anti-VEGF injection / laser therapy	17.17 ± 10.54 months
Çömez, A.	2021	257	Bevacizumab (257)	single injection	0.625 mg/0.025 mL	anti-VEGF injection / laser therapy	121.7 ± 45.7 weeks
Huang, Q.J.	2017	283	Ranibizumab (283)	single injection	0.25 mg/0.025 mL	laser therapy / vitrectomy	at least 6 months.
Hwang, C.K.	22015	22	Bevacizumab (22)	single injection	0.625 mg/0.025 mL	anti-VEGF injection / laser therapy	21.7 ± 8.8 months
Jin, E. Z.	2022	48	Ranibizumab (28)<br>Conbercept (20)	single and multiple injecions	Ranibizumab: 0.25 mg/0.025 mL<br>Conbercept: 0.25 mg/0.025 mL	NA	Ranibizumab: 42.9 ± 9.8weeks<br>Conbercept: 52.6 ± 21.4 weeks
Mintz-Hittner,H.A.	2011	150	Bevacizumab (150)	single injection	0.625 mg/0.025 mL	NA	54 weeks
Lundgren, P.△	2021	29	Bevacizumab (29)	single injection	0.4–0.625 mg<br>(multiple doses)	anti-VEGF injection<br>(no more than four times)	Followed until complete retinal vascularisation or until spontaneous or post-treatment regression of ROP
Bazvand, F.	2021	865	Bevacizumab (865)	single injection	0.625 mg/0.025 mL	anti-VEGF injection / laser therapy	289.4 ± 257 days

Hejkal, T.W.	2025	123	Bevacizumab (123)	single injection	0.03 mg/0.03 mL	NA	4.6 ± 2.8 months
Yetik, H.	2015	238	Bevacizumab (238)	single injection	0.625 mg/0.025 mL	anti-VEGF injection	89.2±4.3 weeks
Akıdan, M.	2026	549	Bevacizumab (261)<br>Ranibizumab (120)<br>Aflibercept (168)	single injection	Bevacizumab: 0.625 mg/0.025 mL	anti-VEGF injection / laser therapy	at least 36 months
Wallace, DK.	2018	58	Bevacizumab (58)	single injection	Ranibizumab: 0.25mg/0.025 mL	anti-VEGF injection / laser therapy	6 months
Murakami, T.	2021	24	Bevacizumab (24)	single injection	Aflibercept: 0.25mg/0.025 mL	anti-VEGF injection / laser therapy	5 years
Chen, SN.	2015	72	Bevacizumab (41)<br>Ranibizumab (31)	single injection	0.25 mg/0.125 mg/0.063 mg/0.031 mg	anti-VEGF injection / laser therapy	52 weeks

*** As follow-up duration varied across studies and it was difficult to unify the time units during data extraction, data were extracted and recorded exactly as reported in the original articles.**

△ **The original research only documented patient counts. There were no specific eye numbers, and the values presented here refers to the number of patients.**

Supplementary Table S3. Clinical outcomes after treatment for ROP patients in 27 included studies.

First Author	Number of Therapied Eyes	Number of Eyes with RD After Treatment	Reactivation / Recurrence, n (%)	The Defination of Reactivation/Recurrence
Wu, W. C. (2013)	162	3	19 (11.7)	The reappearance of plus disease, the persistence or reappearance of neovascularization arising from the retinal vessels, and progression to retinal detachment.
Kang, H.G. (2018)	153	1	15 (9.8)	Requiring additional treatment
Jang, J. H. (2024)	43	1	10 (23.3)	A reactivated stage 2 + or greater lesion at the original site or at the advanced edge
Xu, L. T. (2021)	211	5	23 (10.9)	1) recurrent plus, or 2) recurrent stage 3, or 3) onset of stage 4 or 5 ROP at any PMA, or 4) any other need for repeat anti-VEGF or laser prior to 50 weeks PMA
Bassiouny, Rm. (2021)	216	0	5 (2.3)	Recurrent plus disease, recurrent neovascularization at initial or new advancing ridge despite treatment
Linghu, D. (2022)	1224	34	325 (26.6)	Plus disease or ridge reappearance.
Kabatas, E. U. (2017)	36	0	4 (11.1)	Recurrent plus or preplus disease, progression of traction or recurrent neovascularization.
Erol, M.K. (2015)	36	0	6 (16.7)	Increased arterial tortuosity and venous dilation, formation of retinal neovascularization, and stage III ROP.
Ekinci, D. Y. (2021)	74	3	19 (25.7)	There was fibrovascular proliferation with plus disease after a period of resolution following injection.
Bai, Y. C. (2019)	48	0	8 (16.7)	The recurrence of ridge or plus disease.
Barry, G.P. (2021)	164	1	27 (16.5)	NA
Ling, K. P. (2020)	279	3	33 (11.8)	An initial positive response but later deterioration in retinal status, such as reappearance or worsening of plus disease, neovascularization, and requiring re-treatment.
Nicoară, S. D. (2015)	74	2	11 (14.9)	The persistence/reappearance of plus disease and of retinal neovascularization and the progression toward retinal detachment.

Tong, Q. Z. (2018)	160	35	82 (51.3)	The reappearance of plus disease or the appearance of a ridge
Çömez, A. (2021)	257	1	38 (14.8)	Recurrence of plus disease and extraretinal fibrovascular proliferation, despite initial regression post injection.
Huang, Q. J. (2017)	283	11	127 (44.9)	Eyes with reoccurrence of plus disease or ridge during follow-up.
Hwang, C. K. (2015)	22	0	3 (13.6)	Any of the following: recurrent plus disease, recurrent neovascularization, or progression of traction in spite of treatment.
Jin, E. Z. (2022)	48	0	10 (20.8)	The recurrence of retinal abnormality such as ridge and plus disease after the regression of them.
Mintz-Hittner,H. A. (2011)	150	2	6 (4.0)	Recurrence of retinopathy of prematurity in one or both eyes requiring retreatment before 54 weeks' postmenstrual age
Lundgren, P.* (2021)	29	2	16 (55.2)	Recurrent treatment-requiring ROP after initial regression
Bazvand, F. (2021)	865	17	20 (2.3)	Disease progression
Hejkal, T. W. (2025)	123	0	8 (6.5)	A recurrence of stage 2 disease within zone 2, recurrence of stage 3 in any zone, or recurrence of plus disease in any zone.
Yetik, H. (2015)	123	0	8 (6.5)	NA

*** The original research only documented patient counts. There were no specific eye numbers, and the values presented here refers to the number of patients.**
